# Illuminating and Radiosensitizing Tumors with 2DG-Bound Gold-Based Nanomedicine for Targeted CT Imaging and Therapy

**DOI:** 10.3390/nano13111790

**Published:** 2023-06-02

**Authors:** Maharajan Sivasubramanian, Chia-Hui Chu, Yu Hsia, Nai-Tzu Chen, Meng-Ting Cai, Lih Shin Tew, Yao-Chen Chuang, Chin-Tu Chen, Bulent Aydogan, Lun-De Liao, Leu-Wei Lo

**Affiliations:** 1 Institute of Biomedical Engineering and Nanomedicine, National Health Research Institutes, Zhunan 35053, Taiwan; 2 Department of Biological Science and Technology, China Medical University, Taichung 406040, Taiwan; 3 Department of Cosmoceutics, China Medical University, Taichung 40402, Taiwan; 4 Department of Radiation Oncology, Taipei Medical University Hospital, Taipei 110301, Taiwan; 5 Department of Radiology, The University of Chicago, Chicago, IL 60637, USA; 6 Department of Radiation and Cellular Oncology, The University of Chicago, Chicago, IL 60637, USA

**Keywords:** gold nanodot, 2-deoxy-d-glucose, computed tomography, radiotherapy, contrast agent

## Abstract

Although radiotherapy is one of the most important curative treatments for cancer, its clinical application is associated with undesired therapeutic effects on normal or healthy tissues. The use of targeted agents that can simultaneously achieve therapeutic and imaging functions could constitute a potential solution. Herein, we developed 2-deoxy-d-glucose (2DG)-labeled poly(ethylene glycol) (PEG) gold nanodots (2DG-PEG-AuD) as a tumor-targeted computed tomography (CT) contrast agent and radiosensitizer. The key advantages of the design are its biocompatibility and targeted AuD with excellent sensitivity in tumor detection via avid glucose metabolism. As a consequence, CT imaging with enhanced sensitivity and remarkable radiotherapeutic efficacy could be attained. Our synthesized AuD displayed linear enhancement of CT contrast as a function of its concentration. In addition, 2DG-PEG-AuD successfully demonstrated significant augmentation of CT contrast in both in vitro cell studies and in vivo tumor-bearing mouse models. In tumor-bearing mice, 2DG-PEG-AuD showed excellent radiosensitizing functions after intravenous injection. Results from this work indicate that 2DG-PEG-AuD could greatly potentiate theranostic capabilities by providing high-resolution anatomical and functional images in a single CT scan and therapeutic capability.

## 1. Introduction

According to the World Health Organization (WHO), cancer is the leading cause of death globally, with 10 million deaths in the year 2020 [1]. Several therapeutic strategies are currently in use for the treatment of cancer, such as surgery, radiotherapy (RT) [2,3], chemotherapy [4], immunotherapy [5,6], etc. Among them, RT is considered an important therapeutic intervention. RT uses high energy X-rays or gamma rays to eliminate the tumor through direct and indirect mechanisms [7]. In RT, reactive oxygen species (ROS) are produced by the radiolysis of water, which is lethal to cancer cells by damaging their DNA, thus leading to cell death. ROS are short-lived, highly reactive molecules capable of damaging cellular structures [8]. Although promising, RT presents the major limitation that it has deleterious side effects on healthy tissues [9]. Therefore, an agent which can simultaneously perform targeted imaging and RT functions in cancer is urgently needed. In addition, anatomical imaging modalities, such as X-ray computed tomography (CT), possess excellent spatial resolution when compared to others [10,11,12,13]. Besides tumor targeting, NP can also accumulate in the tumor through passive targeting called the enhanced permeability and retention effect (EPR). However, there is controversy surrounding the EPR effect [14].

CT is often used in RT planning, making it more important than other imaging modalities in RT. Even though CT scans provide markedly better spatial resolution for tumor detection and definition as compared to single photon emission computed tomography (SPECT)/positron emission tomography (PET), it remains insufficient for early diagnosis of cancer where regular scans often miss small premalignant lesions [15]. Currently, clinical CT scanners offer a spatial resolution on the order of ~1 mm. Therefore, an efficient and biocompatible contrast agent that significantly enhances the spatial resolution of CT would be ideally used for early detection and subsequent precision radiation therapy of cancer [16]. Iodine-bound organic molecules are currently being utilized as a commercial CT contrast agent; however, such an approach is constrained by a lack of targeting, poor in vivo circulation, a higher number of iodine atoms per molecule that must be delivered, and imaging must be performed immediately after their administration [17,18,19]. Samei et al. encapsulated an iodinated contrast agent inside a liposome to improve image quality in the blood vessels of breast cancer, but it still presents the disadvantages of causing allergic reactions and toxicity to the kidneys [20]. Vast developments in material science and technology have enabled scientists to create theranostic nanoparticles that integrate diagnostic and therapeutic functions in a single nanoparticle entity. These multifunctional nanoplatforms have great potential in clinics for two main reasons: (1) They can assist clinicians and physicians to gather more information to plan and execute therapy; (2) They allow monitoring of drug release, prediction of treatment outcome, and classification of patients into subpopulations based on characteristics of tumor. Indeed, in the near future, theranostic nanoparticles will facilitate the transformation of general cancer treatments towards personalized cancer care [21].

Gold nanomaterials are attractive for use in medical applications because they are inert, biocompatible, offer ease of surface chemical modification, and possess unique surface plasmon resonance [22]. Synthetic approaches of gold nanoparticles are well investigated in terms of control over their size, morphology, and surface modification [23]. Moreover, gold is a high Z element with a high X-ray absorption coefficient (at 100 keV, the coefficients of gold and iodine are 5.16 and 1.94, respectively). These features encourage the exploitation of gold nanomaterials as potential candidates for CT contrast enhancement [24,25,26,27,28,29,30]. Extant literature also reported positive outcomes as excellent radiosensitizers using gold nanoparticles in radiation therapy [31,32,33,34,35,36]. Therefore, it is conceivable that targeted gold nanoparticles could simultaneously serve the dual purpose of an imaging contrast agent and a radiotherapy dose enhancer. Recently, radiotherapeutic efficacy of gadolinium (Gd) nanoparticles was investigated in a lung tumor-bearing mice model. The results showed that Gd NP were able to suppress the tumor growth significantly after X-ray irradiation and did not induce any toxicity in the healthy tissues of the mice as evidenced by the absence of significant changes in their body weight during the entire treatment period as compared to X-ray only treatment [37].

The goal of this study is to develop and characterize 5 nm gold nanodots (AuD) modified with 2-deoxy-d-glucose (2DG) as a novel targeted therapeutic and CT contrast agent for RT. In a previous study, 2DG-labeled AuD showed enhanced uptake and elevated CT contrast at cellular levels [38,39]. Furthermore, polyethylene glycol (PEG) was introduced to increase water solubility and blood half-life to improve its imaging applicability [40]. AuD was sequentially conjugated with PEG and 2DG, termed 2DG-PEG-AuD, to enhance its preferential uptake by cancer cells. 

Considering the small size of AuD (5 nm), we hypothesize that the cell uptake mechanism of 2DG-PEG-AuD might be similar to that of [^18^F]DG used in PET. 2DG is a glucose derivative, with the hydroxyl group at the 2-carbon position replaced by hydrogen so that the phosphorylated 2DG by glucose hexokinase is trapped within the cell and cannot be further metabolized through the subsequent steps of glycolysis. The accumulation of 2DG in the cell might contribute to the underlying mechanism of CT contrast enhancement by our synthesized 2DG-PEG-AuD. To determine the feasibility of clinical translation, tumor-bearing mouse models were employed to evaluate the in vivo ability of 2DG-labeled AuD as a CT contrast agent. Since many tumor cell types exhibit a high level of glucose uptake and elevated hexokinase expression, 2DG-PEG-AuD may possess great potential for use as an efficient radiosensitizer and CT contrast agent for precise cancer detection and therapy.

## 2. Materials and Methods

### 2.1. Materials

Chloroauric acid (HAuCl_4_, 99.9%), potassium carbonate, tannic acid, sodium citrate dehydrate, 2-amino-2-deoxy-d-glucose hydrochloride, 2-deoxy-d-glucose, 1-ethyl-3-(3-dimethylaminopropyl) carbodiimide (EDC), doxorubicin (DOX), osmium tetroxide (OsO_4_, 99.8%), methylene blue (MB), Diphenylbenzofuran (DPBF), dihydroethidium (DHE), and Spurr resin were all purchased from Sigma-Aldrich (Darmstad, Germany). Thiol carboxylic polyethylene glycol (SH-PEG-COOH, 5K) and thiol polyethylene glycol (SH-PEG, 5K) were purchased from Nanocs (Boston, MA, USA). Vivaspin^®^ 20 centrifugal concentrators were obtained from Sartorius (Goettingen, Germany).

### 2.2. Characterization Methods

Morphologies of the samples were characterized via transmission electron microscope (TEM, Hitachi, Chiyoda, Tokyo, Japan, H-7650 operating at an acceleration voltage of 80 kV). Steady-state absorption spectra were acquired using a DU800 UV spectrometer (Beckman, Spectral lab Scientific Inc., Markham, ON, Canada) and Cary Eclipse Fluorescence spectrometer (Varian, Palo Alto, CA, USA). CT imaging was performed using a micro CT scanner (TriumphTM X-OTM, Gamma Medica-Ideas Inc., Northridge, CA, USA). The images were acquired at 75 kVp, 135 μA with a 512 × 512 matrix size and 360 views over a full circle. Image reconstruction was performed using a proprietary filtered backprojection algorithm implemented in the imaging system’s on-board reconstruction software (Cobra, Exxim Computing Corporation, Pleasanton, CA, USA). The reconstructed image volume consisted of 512 × 512 × 512 volume elements with an isotropic size of size of <60 µm. Samples were irradiated by using X-ray irradiator (RS-2000, Rad Source Co., Ltd., Buford, GA, USA).

### 2.3. Synthesis of Gold Nanodots (AuD)

Spherical AuD were synthesized in aqueous solutions using sodium citrate and tannic acid as reducing and stabilizing agents, respectively [35]. To attain 30 mg of AuD, we started with solution a (5.18 mL of 1% (*w*/*v*) HAuCl_4_) and solution b (20.72 mL of 1% (*w*/*v*) trisodium citrate dehydrate), 25.9 mL of 1% tannic acid, and 25.9 mL of 2.5 mM K_2_CO_3_. Both solutions a and b were heated to 60 °C using a water bath and mixed while stirring rapidly. The mixed solution was boiled for 10 min and then cooled to room temperature. The size of AuD was checked using UV-Vis spectroscopy. The resonance spectrum showed peaks between 520 to 525 nm. The products were then centrifuged using Vivaspin^®^ 20 (Sartorius, Germany) centrifugal concentrators to remove the unreacted precursors and water. Subsequently, AuD products were stored in the dark at 4 °C.

### 2.4. Synthesis of 2DG-PEG-AuD

Two types of PEG chains were used to modify the surface of AuD: SH-PEG-COOH and SH-PEG. First, 20 moles of SH-PEG-COOH were mixed and stirred with one mole of AuD for 2 h, followed by the addition of 200 moles of SH-PEG to the mixture to cover the entire surface of AuD. Free PEG chains were washed and removed by using 30 kDa Vivaspin^®^ 20 centrifugal concentrators. EDC was then added into the PEGylated AuD and stirred for 2 h at 4 °C. The product was centrifuged using 30 kDa Vivaspin^®^ 20 centrifugal concentrators to remove the free reactants. The mole ratio of AuD:2-amino-2-deoxy-d-glucose hydrochloride:EDC is 1:200:2000.

### 2.5. In Vitro CT Imaging and Reactive Oxygen Species (ROS) Formation Analysis

AuD samples with a serial concentration of 1, 3, 5, 10, 30, 60, and 80 mg/mL were imaged using a microCT scanner. A vial of water was also included in the CT scan as a reference. Due to their biological significance, we quantified the formation of hydroxyl (•OH), singlet oxygen (^1^O_2_), and superoxide radicals (•O_2_^−^). We employed MB (absorbance at 660 nm), DPBF (excitation: 414 nm and emission: 460 nm), and DHE (excitation: 490 nm and emission 585 nm) as the probes for the detection and quantification of •OH, ^1^O_2_, and •O_2_^−^, respectively. All the ROS probes were premixed with 350 µg of AuD-PEG and the resulting final concentrations of DPBF, DHE, and MB were 40 μM, 10 μM, and 10 mM. Solutions were then exposed to X-rays.

### 2.6. Cytotoxicity Assay

The cytotoxic effect of 2DG-PEG-AuD was determined by standard MTT assay in 96-well plates. Approximately 5 × 10^3^ cells were placed in each well and cultured at 37 °C with 5% CO_2_. After 24 h of incubation, media were removed and replaced with fresh medium with 10% FBS containing 2DG-PEG-AuD at concentrations ranging from 50 µg/mL to 200 µg/mL. Cells were incubated for 24 h, and then the medium containing 2DG-PEG-AuD was replaced with 180 µL of fresh medium, followed by the addition of 20 µL of MTT reagent. After 4 h of culturing, the medium was substituted with DMSO (150 µL). The cell viability was evaluated via absorbance measurements at 570 nm.

### 2.7. In Vitro Cellular Uptake Assay

LS174T, the human colon cancer cell line, was chosen for in vitro cellular uptake assay since studies showed that LS174T overexpressed hexokinase and was demonstrated as [^18^F]DG-avid cell line [28]. LS174T cells were seeded into 12-well plates and incubated with four different conditions: medium alone; PEG-AuD; 2DG-PEG-AuD; and 2DG-PEG-AuD with pretreated 2DG 1 h in advance. After 2 h of treatment, cells were harvested, and CT imaging was performed.

### 2.8. DNA Damage and Clonogenic Assay

DNA damage in LS174T cells after treatment with X-rays and X-rays combined with 2DG-PEG-AuD was assessed by the formation of γ-H2AX foci. Briefly, after different treatments for 24 h, the cells were washed twice with PBS, fixed with 4% glutaraldehyde for 10 min, and permeabilized with 0.5% Triton X-100. Next, cells were blocked in 5% bovine serum albumin for 1 h and subsequently incubated overnight at 4 °C with monoclonal antihuman phospho-H2AX (S139) mouse mAb (Millipore, Burlington, MA, USA) at 1:1000 dilution in PBS (with 0.1% Triton X-100 and 5% BSA). The cell nucleus and membranes were stained with DAPI and WGA594. The appropriate cell numbers were plated for survival analysis. The culture medium was removed and replaced with nanoparticle-containing culture medium for 24 h at 37 °C. Radiation was delivered in a single dose of 0–10 Gy over an appropriate field size at a dose rate of 87.8 mGy s^−1^. After irradiation, the cells were harvested, counted, and seeded on 10 mm dishes. Cell cultures were then incubated for 10–14 d at 37 °C in 5% CO_2_ in air at 95% humidity. Colonies were then fixed with glutaraldehyde (6.0% *v*/*v*), stained with crystal violet (0.5% *w*/*v*), and counted with each experiment performed in quadruplicate.

### 2.9. Western Blot Analysis

Membrane proteins were extracted from LS174T cells using an s compartmental protein extraction kit (Chemicon Inc, Temecula, CA, USA). After the various treatments, LS174T cells were lysed with RIPA lysis buffer supplemented with protease and inhibitor for 30 min on ice followed by centrifugation at 4 °C. The amount of protein was measured by BCA assay (Thermo Fisher Scientific, Waltham, MA, USA) and an equal amount of protein lysates was electrophoresed on an 8–15% polyacrylamide gel at 100 volts for 2 h. The proteins were transferred to a polyvinylidene difluoride (PVDF) membrane (0.2 µm pore size) through wet blotting at 250 mAmp for 2 h (in cold room) (Biorad, Billerica, MA, USA). Blocking was performed using 5% BSA in 1x PBS-T (1X PBS 0.1% TWEEN-20) for 1 h at RT, and then the membrane was incubated with primary antibodies at 4 °C overnight. After that, a washing process was performed four times with 1x PBS-T, and the samples were incubated with secondary antibody HRP–goat–anti-rabbit/mouse (1:10,000) for 1 h at RT. Then, membranes were washed with 1X PBS-T and developed using a Femto chemiluminescent substrate (Thermo Scientific, Waltham, MA, USA) and the image analysis was carried out using ImageJ software. Antibodies for Western blotting, including GPX4 (GTX54095, 1:1000), β actin (GTX109639, 1:5000), were purchased from GeneTex, Irvine, CA, USA.

### 2.10. In Vivo CT Imaging and Transmission Electron Microscopy (TEM)

All experiments that involved animals were performed in accordance with the guidelines of the Management Group of Animal Experiments (MGAE) in Taiwan (NHRI-IACUC-104012-A). In vivo experiments were conducted using five- to six-week-old male nu/nu mice (BioLASCO Taiwan Co., Ltd., Taipei, Taiwan). The mice were anesthetized with isoflurane (1–4% inhalation), and the LS174T cells (4 × 10^6^ cells in 0.2 mL PBS) were subcutaneously injected into right flanks of the mice. CT imaging was performed using the Trimodality preclinical microPET/SPECT/CT imaging system. For animal studies, six-week-old nu/nu mice were inoculated with LS174T cells subcutaneously on the right flank. AuD samples were i.v. injected through the tail vein at a speed of 16 µL/min when the tumor reached 150 to 200 mm^3^. CT imaging was performed 24 h after administration. The images were acquired at 60 kVp, 135 μA, with a 512 × 512 matrix size and 360 views over a full circle. Image reconstruction was performed using a proprietary filtered back projection algorithm implemented in the imaging system’s on-board reconstruction software (Cobra, Exxim Computing Corporation, Pleasanton, CA, USA). The reconstructed image volume consisted of 512 × 512 × 512 volume elements with an isotropic size of <60 μm.

TEM imaging of AuD, PEG-AuD, and 2DG-PEG-AuD particles was carried out using a Libra 120 TEM (Carl Zeiss, Bendemeer Road, Singapore). A carbon-coated 200 mesh copper grid was pre-rinsed with 5 μL of ethanol and then deposited with 5 μL of the suspension of AuD samples in ethanol. After 3 min, the sample was dried with filter paper and further dried with N_2_ gas flow. For TEM imaging of tumor slides, mice were sacrificed after CT imaging, and their tumor specimens were fixed overnight in glutaraldehyde buffered (2.5%) with phosphate buffered saline (PBS; 0.1 M, pH 7.4). Tissues were then washed three times in PBS and post-fixed for 1 h in a solution that contained OsO_4_ buffered (2%) with PBS. Tissues were subsequently washed three times in H_2_O and dehydrated stepwise in ethanol. Tissues were then polymerized using Spurr resin at 68 °C for 15 h and then embedded specimens were sectioned into 70 nm slices and viewed on a Hitachi H-7650 TEM operated at 80 kV.

### 2.11. In Vivo Tumor Oxygen Saturation Measurements by Pa Imaging

In vivo tumor oxygenation saturation (sO_2_) measurements were performed using the PA imaging system. For this purpose, LS174T tumor-bearing mice (150–200 mm^3^) were randomized to four treatment groups: control; 2DG-PEG-AuD (40 mg, i.v. at a speed of 16 µL/min); X-ray (total 8 Gy; four fractions of 2 Gy every day). In the combined treatment group (X-ray + 2DG-PEG-AuD), 24 h post-injection, the tumors in mice were irradiated using an X-ray irradiator (total 8 Gy; four fractions of 2 Gy every day) and PA imaging was performed. Mice from the treatment groups were deeply anesthetized with isoflurane (1–4%) using an inhalation device and placed on a heating pad. A 128-channel Verasonics high-frequency US platform (Vantage 128, Verasonics Inc., Washington, DC, USA) was employed for dual-modality imaging (both PA imaging and US imaging). The entire PA system was controlled by a custom-developed graphical user interface (GUI) based on MATLAB^®^ (R2007a, MathWorks Inc., Natick, MA, USA). To operate the system in the PA mode, laser excitation and data acquisition were synchronized using triggering. The excitation laser was a compact Nd: YAG-laser system with an integrated tunable optical parametric oscillator (OPO, SpitLight 600 OPO, InnoLas Laser GmbH, Bavaria, Germany). The OPO generates approximately 7-ns duration pulses at a 20-Hz repetition rate with tunable wavelengths from 680 to 2400 nm. The PA signals were acquired using a high-frequency 18.5-MHz US transducer (L22-14v, Verasonics Inc., Washington, DC, USA). This transducer has a -6-dB fractional bandwidth of 67% and 128 active elements. The sO_2_ around the tumor was measured using the differential optical absorption of oxygenated and deoxygenated hemoglobin at different wavelengths of 850 and 750 nm, respectively. To facilitate the comparison of sO_2_ patterns in different groups, the regions of interest (ROI) in the whole tumor were employed and identified using US imaging. PA B-scans of the mice generating averagely oxygenated and deoxygenated hemoglobin signals were analyzed using custom-developed software based on MATLAB^®^ (R2007a, The MathWorks, Natick, MA, USA), and sO_2_ is defined as sO_2_ = [HbO_2_]/[HbO_2_] + [Hb].

### 2.12. In Vivo Therapeutic Efficacy

The tumors were allowed to reach approximately 150 to 200 mm^3^ in volume estimated with the formula 1/2 (L × W^2^), where L and W were the length and width, respectively. The mice were randomized to four treatment groups: control; 2DG-PEG-AuD (40 mg, i.v. at a speed of 16 µL/min); radiation (total 8 Gy; four fractions of 2 Gy every day); and a combination group. In the combined treatment group, after 24 h post-injection, the tumors in mice were irradiated using an X-ray. Tumor volume observations were assessed using a caliper twice per week. For each group, histological analyses were performed on tumors collected after tumor size tracing.

## 3. Results and Discussion

**Figure 1:**
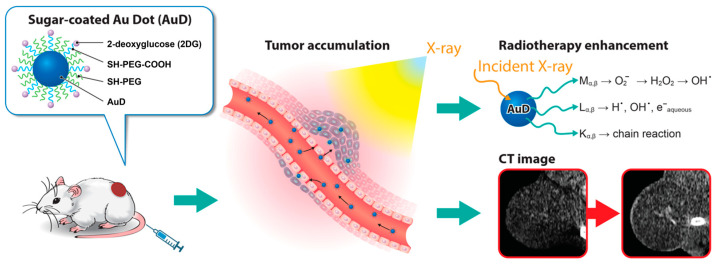
Schematic illustration of 2DG-PEG-AuD synthesis.

**Figure 2:**
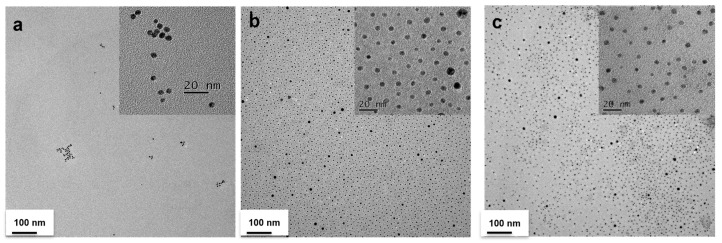
TEM images of (**a**) AuD, (**b**) PEG-AuD, and (**c**) 2DG-PEG-AuD.

AuD 5 nm in diameter were synthesized by citric acid reduction of chloroauric acid and coated with a high molecular weight of PEG (5000 Da) for in vivo circulatory enhancement and then conjugated with 2-DG to derive 2DG-PEG-AuD. The schematic synthesis of 2DG-PEG-AuD is shown in Figure 1. We employed two types of PEG chains: SH-PEG-COOH and SH-PEG. The ratio of SH-PEG-COOH and SH-PEG was empirically optimized and fixed at 10. Carboxylic acid groups of SH-PEG-COOH were used to link the amine group of 2-amino-2-deoxy-d-glucose hydrochloride to form stable amide bond crosslinks. Figure 2 presents the morphology of AuD with a uniform size of approximately 5 nm in diameter measured with TEM and an average diameter of 11.6 nm using dynamic light scattering (DLS, Nano ZS Zetasizer, Malvern, UK) analysis. After conjugation with PEG, PEG-AuDs were well dispersed, as shown in the TEM image, and the median size increased to 14 nm as measured with DLS, indicating the successful conjugation of PEG. With 2DG conjugation, 2DG-PEG-AuD still exhibited excellent dispersity in the TEM image, and the size increased to 20 nm as analyzed with DLS. Zeta-potential data display the surface charge change from −31 mV to −24 mV after PEGylation (Table 1), which might be ascribed to the citrate acid and tannic acid replacement from the surface of AuD. After 2DG conjugation, zeta-potential changed from −24 mV to −11 mV due to the substitution of carboxylic acid for 2DG.

**Figure 3:**
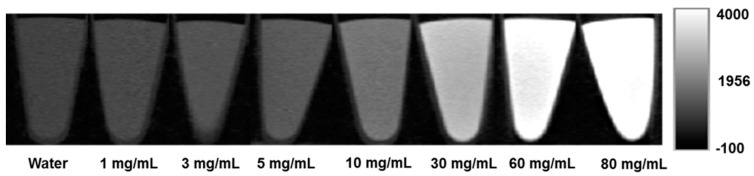
Coronal slices of CT images of AuD suspension at different concentrations.

In order to evaluate the contrast-producing property of AuD and to establish a quantitative relationship between the concentration of the AuD suspension and the resultant CT contrast, AuD samples with a serial concentration of 1, 3, 5, 10, 30, 60, and 80 mg/mL were imaged using a micro CT scanner. A vial of water was also included in the CT scan as a reference. The coronal slices of these CT images are shown in Figure 3 and the corresponding HU units are provided in Table 2. The results showed a bright CT contrast in a dose-dependent manner. In addition to CT contrast enhancement, we investigated RT efficacy by evaluating the formation of biologically important cytotoxic ROS.

•OH is a highly reactive and unstable molecule that is produced during RT. The radiation interacts with water molecules in the cells of the tumor and surrounding tissue, leading to the formation of •OH. They can then react with DNA, proteins, and other molecules in the cells, causing damage and ultimately leading to cell death. The production of •OH during radiation therapy is a key mechanism through which the treatment works to kill cancer cells [41].

**Figure 4:**
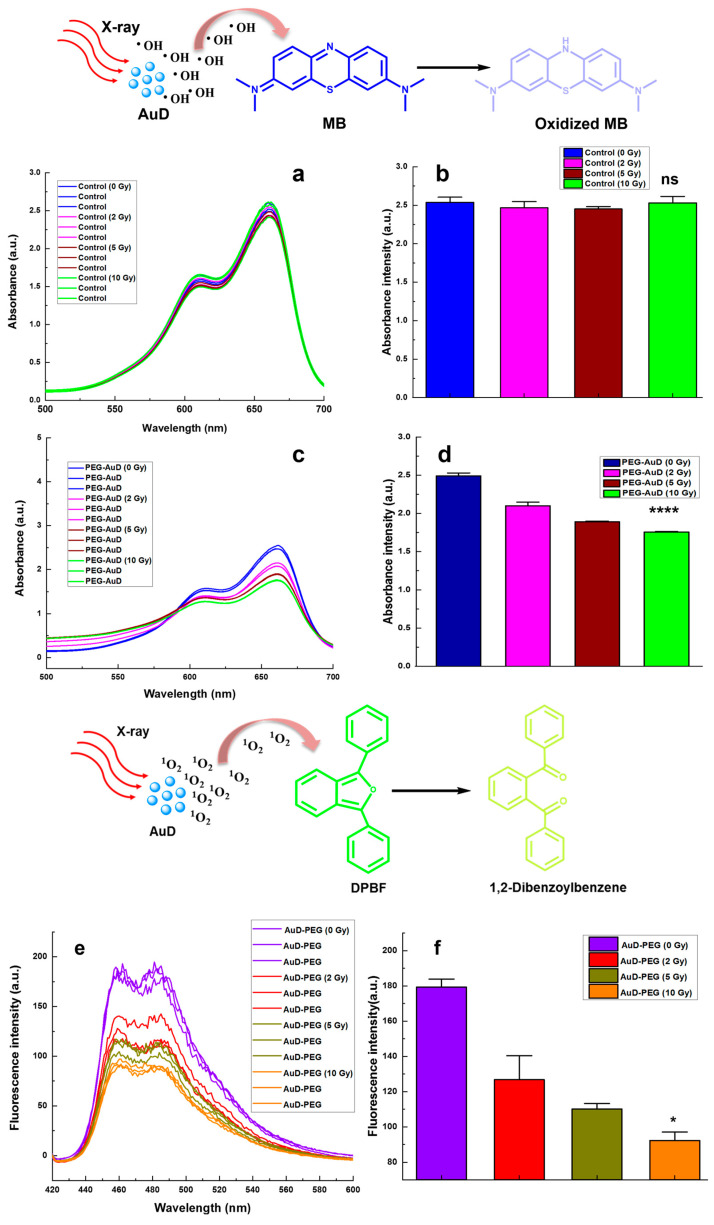

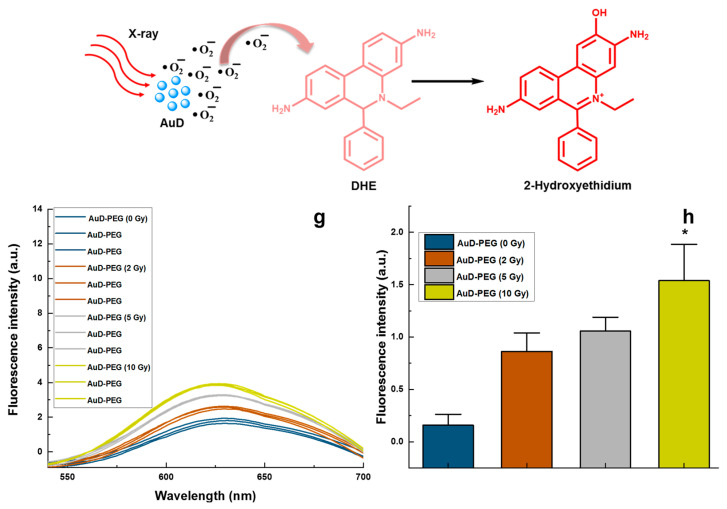
•OH, ^1^O_2_, and •O_2_^−^ formation analysis (**a**) Absorbance spectrum of MB in water irradiated with various X-ray doses and their quantification (**b**,**c**) Absorbance spectrum of MB combined with PEG-AuD in water irradiated with various X-ray doses and their quantification (**d**–**f**) Fluorescence spectrum of DPBF combined with PEG-AuD in water/DMSO mixture irradiated with various X-ray doses and their quantification (**g**,**h**) Fluorescence spectrum of DHE combined with PEG-AuD in water irradiated with various X-ray doses and their quantification. Asterisks (*, ****) denote statistically significant differences (*p* < 0.05, *p* < 0.0001) compared with X-ray combined AuD-PEG samples and ns denotes non-significant.

To investigate the formation of •OH, we employed MB as a probe. MB is oxidized by •OH, characterized by their decrease in the absorbance at 660 nm. Figure 4a,b show the visible spectrum of the control group (MB + water) with or without X-ray; clearly there was no significant change in MB absorbance. Even the highest dose of 10 Gy did not induce a noticeable change in MB absorbance, which indicates much less formation of •OH. Whereas the combination group of PEG-AuD + MB + X-ray (Figure 4c,d) showed a dose dependent decrease in MB absorbance, PEG-AuD + MB without X-ray maintained the base level absorbance the same as the control group; this clearly indicates that AuD is crucial for the formation of •OH. The absorbance of MB decreased with the increase in the X-ray dose; this might be due to the sensitization of X-rays to the AuD, which led to the formation of copious amounts of •OH. For the analysis of ^1^O_2_ and •O_2_**^−^** formation, the control group (ROS indicator + X-ray) was not included due to the vast changes in fluorescence intensity between the control and the PEG-AuD (14 nm) group caused by the latter’s interference affecting the fluorescence spectrum of ROS indicators. Figure 4e,f show the effect of PEG-AuD on the enhanced generation of ^1^O_2_ production under various X-ray doses; it was quantified by measuring the decrease in the DPBF fluorescence at 460 nm. The photobleaching of DPBF in the presence of PEG-AuD as a function of X-ray dose can be clearly seen, as compared to DPBF + PEG-AuD without X-ray irradiation (0 Gy). The observed decrease in DPBF fluorescence with X-ray dose in the presence of PEG-AuD shows the efficient generation of highly reactive ^1^O_2_. Figure 4g,h show the fluorescence spectrum of DHE after treatment with PEG-AuD combined with various X-ray doses. The fluorescence of DHE increased with the X-ray dose, which denotes the formation of •O_2_^−^ which converted DHE to a highly fluorescent 2-hydroxy ethidium.

**Figure 5:**
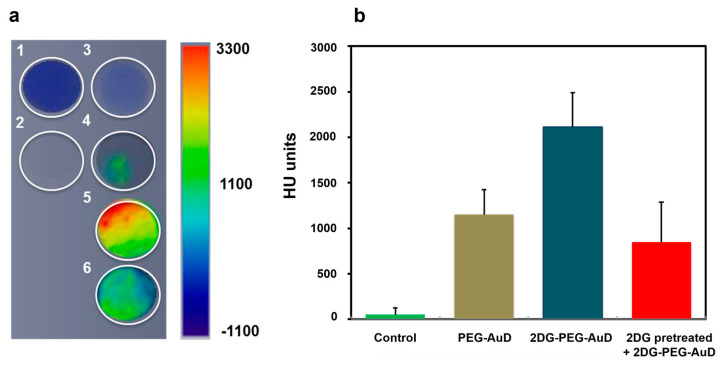
(**a**) Axial CT slices of cell samples. Cells incubated with various formulations and collected in Eppendorf vials for CT images. The color bar on the right represents the intensity of CT contrast. (1) Water, (2) air, (3) control cells, (4) cells with PEG-AuD, (5) cells with 2DG-PEG-AuD, and (6) 2DG pretreated cells with 2DG-PEG-AuD. (**b**) Quantification of CT contrast.

**Figure 6:**
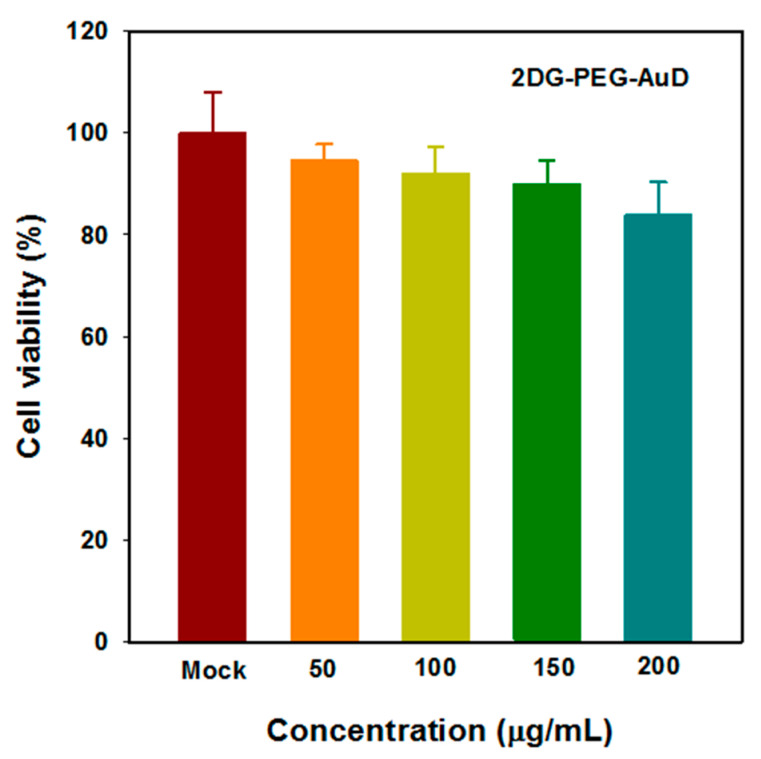
Cell viability of LS174T cells incubated with 2DG-PEG-AuD under various concentrations.

To demonstrate the efficient internalization of 2DG-PEG-AuD by cancer cells, we employed LS174T, a human colon cancer cell^−^ line, that was reported with [^18^F]DG positive response and highly expressed hexokinase, as a cell study model. LS174T cells were seeded in triplicate for each treatment: serum free medium alone; PEG-AuD; and 2DG-PEG-AuD. In the competition study, we pretreated cells with 2DG for 1 h to occupy the glucose transferase, followed by the addition of 2DG-PEG-AuD. After 2 h of incubation, cells were washed and trypsinized into vials. For CT imaging data acquisition, one vial of water and one empty vial (air) were also performed as a reference. Axial CT slides of cell samples are presented in Figure 5a, and the quantification results are shown in Figure 5b. Cells treated with 2DG-PEG-AuD (with a concentration of 200 µg/mL) showed strong CT contrast compared to the other treatment groups. This indicates efficient uptake by the cells due to the targeting by 2DG, while PEG-AuD-treated cells showed low uptake and weak CT contrast due to the lack of targeting. CT contrast decreased for the cells pre-treated with 2DG, which binds to the hexokinase that limits the uptake of 2DG-PEG-AuD. The concentration of 2DG-PEG-AuD (200 µg/mL) does not induce toxicity to LS174T cells, as demonstrated in Figure 6. With 2DG-PEG-AuD, cell internalization increased 1.8 times when compared to that of PEG-AuD. In addition, pretreating LS174T cells with free 2DG for 1 h significantly inhibited the cell uptake of 2DG-PEG-AuD, implicating the same pathway for cell internalization of 2DG and 2DG-PEG-AuD.

We then evaluated the cytotoxic effects of 2DG-PEG-AuD on LS174T cells by MTT assay (Figure 6). Cell viability was retained above 80% with the tested nanodot concentrations ranging from 50 to 200 µg/mL. However, due to their biocompatible nature, in all of the tested concentrations, cell viabilities were retained over 80%; this indicates that the components of our nanoplatform were not harmful to cancer cells.

**Figure 7:**
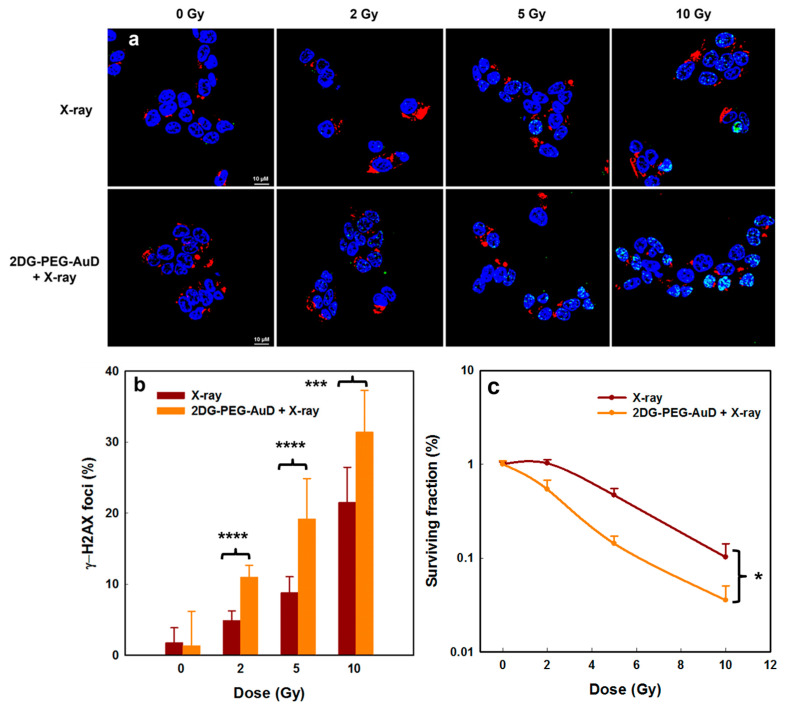
(**a**) Immunofluorescent imaging of LS174T cells incubated with or without 2DG-PEG-AuD under various X-ray irradiation doses. Blue fluorescence indicates the nucleus, stained by DAPI, green fluorescence indicates γ-H2AX foci and red fluorescence indicates cell membrane, stained by WGA594. Scale bar is 10 µm. (**b**) Quantification of γ-H2AX foci and (**c**) clonogenic survival assays of 2DG-PEG-AuD-incubated LS174T cells for 24 h followed by irradiation with various doses of X-rays. Asterisks (*, *** and ****) denote statistically significant differences (*p* < 0.05, *p* < 0.001, and *p* < 0.0001) compared with X-ray only samples.

The DNA double strand break (DSB) is the lethal damage caused by RT resulting in cell death. Phosphorylation of the histone H2AX (γ-H2AX foci), recognized as the early stage of DSB in cell nuclei, was observed by confocal microscopy. To assess the in vitro radiotherapy performance, we exposed the 2DG-PEG-AuD-treated cells to various doses of X-ray and compared with X-ray-only-treated cells. In the 2DG-PEG-AuD-treated cells, as shown in Figure 7a, the formation of γ-H2AX foci in the DNA can be clearly seen as green fluorescence, denoting DNA damage irrespective of X-ray dose. Interestingly, the green fluorescence of γ-H2AX increased with X-ray dose, and NP-treated cells with 10 Gy X-ray exposure generated the highest level of foci formation. In the X-ray-only-treated cells, however, the level of DNA damage induced was low compared to that in the 2DG-PEG-AuD-treated cells. The fluorescence quantitative analysis is shown in Figure 7b. The γ-H2AX foci percentage in the 2DG-PEG-AuD-treated group was higher than that in the control. Moreover, an X-ray dose-dependent increase in the foci was observed, and the highest foci percentage was seen in the 10 Gy-irradiated group. From these results, the presence of 2DG-PEG-AuD exacerbated DNA damage with increasing dose, which implies its potential as a radiosensitizer.

The proliferative potential of cancer cells can be evaluated by clonogenic assay since X-ray irradiation induces slow apoptosis and can reduce cell proliferation within several days. When LS174T cells were incubated with 2DG-PEG-AuD and then exposed to 2, 5, and 10 Gy of X-ray irradiation, the survival fraction was found to be lower than that of X-ray irradiation alone at all doses. X-ray irradiation treatment at 10 Gy alone induced only 10.02% of colony formation, while 2DG-PEG-AuD combined with X-ray treatment at 10 Gy, ultimately led to 7% of colony formation (Figure 7c). These results suggested that 2DG-PEG-AuD indeed has the potential to act as a radiosensitizer to enhance RT.

Then, we investigated the mechanism of cell death in the presence of 2DG-PEG-AuD combined RT. Ferroptosis is a form of regulated cell death that is characterized by the accumulation of lipid peroxidation and iron-dependent ROS generation. It is distinct from other forms of cell death, such as apoptosis, necrosis, and autophagy. One of the key regulators of ferroptosis is glutathione peroxidase 4 (GPX4), an enzyme that is responsible for reducing lipid hydroperoxides to their corresponding alcohols, thereby preventing lipid peroxidation and subsequent cell death. GPX4 uses reduced glutathione as a cofactor to reduce lipid peroxides. When GPX4 is inhibited, either by genetic or pharmacological means, lipid peroxidation accumulates, leading to ferroptosis [42].

**Figure 8:**
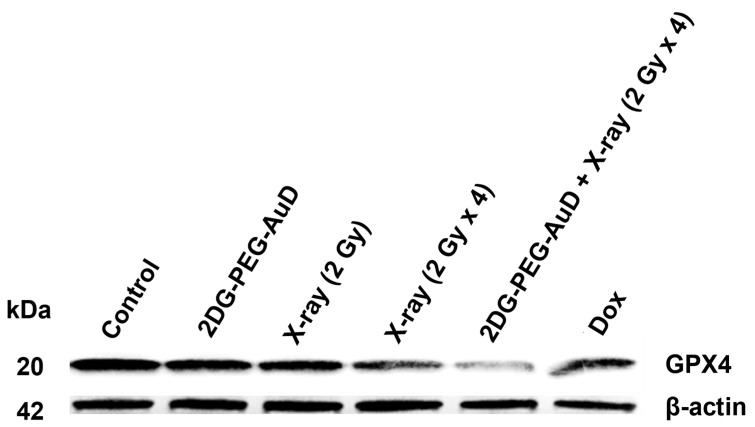
Western blot analysis of the expression levels of GPX4 by LS174T cells under various treatments.

DOX used as a positive control slightly lowered the expression levels of GPX4. Compared to the control, both 2DG only and X-ray (2Gy) groups showed GPX4 expression levels similar to the control group. The X-ray (2Gy × 4) group clearly showed a noticeable decrease in the expression of GPX4, which indicates the onset of ferroptosis. Interestingly, in the combination group 2DG-PEG-AuD + X-ray (2Gy × 4), the expression levels of GPX4 were significantly reduced compared to the rest of the treatment groups. The observed trend could be explained by the generation of copious •OH, which fuels the peroxidation of phospholipids promoting ferroptosis (Figure 8).

**Figure 9:**
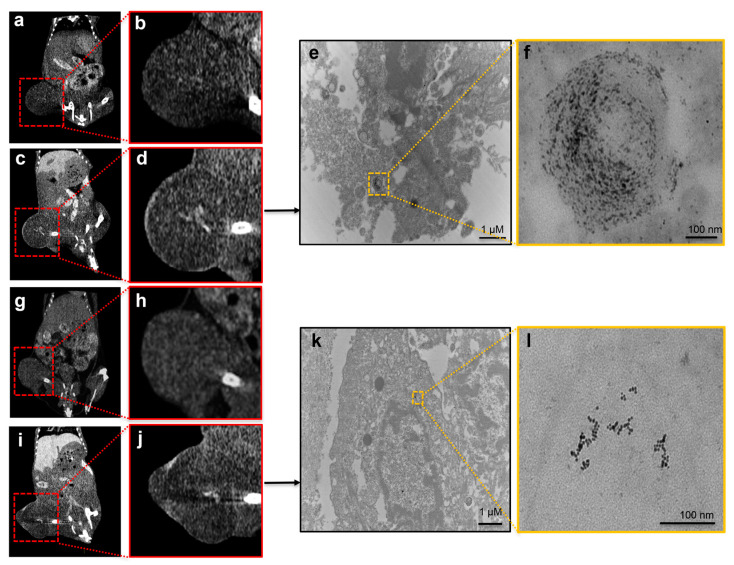
In vivo CT images of tumor-bearing mice with 2DG-PEG-AuD/PEG-AuD administration through i.v. injections. Red boxes delineate the regions of the tumors. CT images of tumors were scanned before administration (**a**,**b**,**g**,**h**) and after 2DG-PEG-AuD (**c**,**d**) and PEG-AuD (**i**,**j**) injections. TEM images of tumor sections were acquired from mice treated with 2DG-PEG-AuD (**e**) and PEG-AuD (**k**). (**f**,**l**) are magnified images of regions of interest outlined by yellow boxes in (**g**) and (**h**), respectively.

The xenograft LS174T tumor-bearing mouse model was used to validate the in vivo capability of 2DG-PEG-AuD used as a CT contrast agent. When the tumors reached the sizes of 150 to 200 mm^3^, various AuD samples were i.v. administered through tail veins at a speed of 16 µL/min. CT scans were performed 24 h post-administration. After CT scans, tumors were harvested and sectioned into 70 nm slices for TEM images. Figure 9 presents CT images before (a, d) and after (b, e) the administration of 2DG-PEG-AuD. CT contrast enhancement at the tumor site, pronounced at the peripheral region (Figure 9e), was observed in comparison with the pre-injection scan (Figure 9d). The heterogeneous distribution of 2DG-PEG-AuD accumulation in the tumor possibly resulted from the high pressure and pressure gradients in the tumor tissue and/or the necrosis-associated disintegration of blood vasculature that occurred predominantly at the central tumor. TEM images of the tumor section from mice treated with 2DG-PEG-AuD showed remarkable accumulation of AuD (black dots in yellow box) inside of the cells. In contrast, mice treated with PEG-AuD showed little accumulation (Figure 9h,j) and markedly less contrast enhancement in CT images (Figure 9c,f).

**Figure 10:**
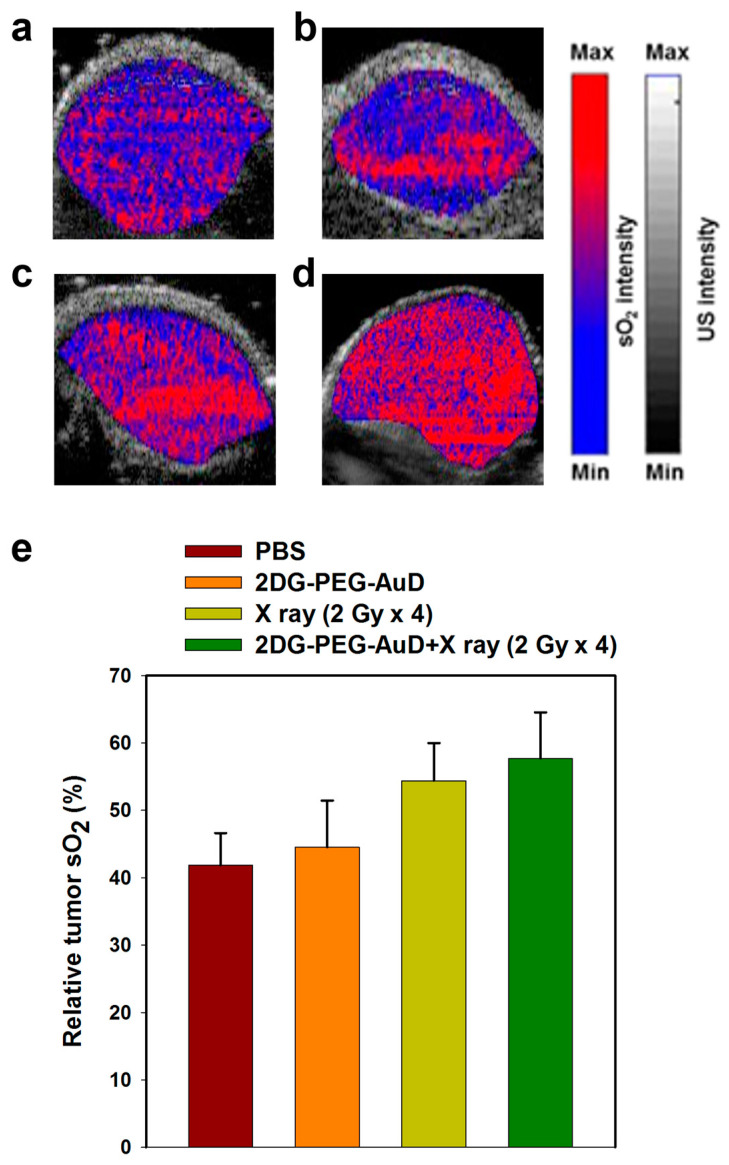
PA tumor sO_2_ images after the treatment of (**a**) PBS, (**b**) 2DG-PEG-AuD, (**c**) X-ray (2Gy × 4), (**d**) 2DG-PEG-AuD + X-ray (2Gy × 4), (**e**) quantitative PA tumor sO_2_ values.

The efficacy of radiotherapy strongly depends on the tumor oxygen tension. Fractionated radiotherapy can influence tumor oxygen saturation by allowing time for the re-oxygenation of tumor cells between radiation treatments. When radiation is delivered in smaller doses over a period of time, there is more time for oxygen to be replenished in the tumor cells between treatments. This can help improve the effectiveness of radiation therapy against cancer cells by increasing the amount of well-oxygenated cells in the tumor. Using PAI,we examined tumor sO_2_ maps after various treatments as shown in Figure 10a–e. Both control and 2DG-PEG-AuD treatment showed no significant difference between them in the sO_2_ map; sO_2_% was calculated to be ~42% and ~45% for PBS and 2DG-PEG-AuD. However, an obvious and notable increase in the red signal was observed in tumor sO_2_ for the X-ray (2Gy × 4) treatment group, which could be ascribed to the re-oxygenation of tumors between the intervals of radiation treatment. Compared to the control group, a 55% increase in tumor sO_2_ for the X-ray (2Gy × 4) treatment group was found. Among the all treatment groups, a combination of 2DG-PEG-AuD + X-ray (2Gy × 4) showed the highest increase in the tumor sO_2_ (65%), evidenced by the increase in the red signal in the tumor sO_2_ map. The observed increase in tumor sO_2_ after fractionated radiotherapy is a reported phenomenon [43].

**Figure 11:**
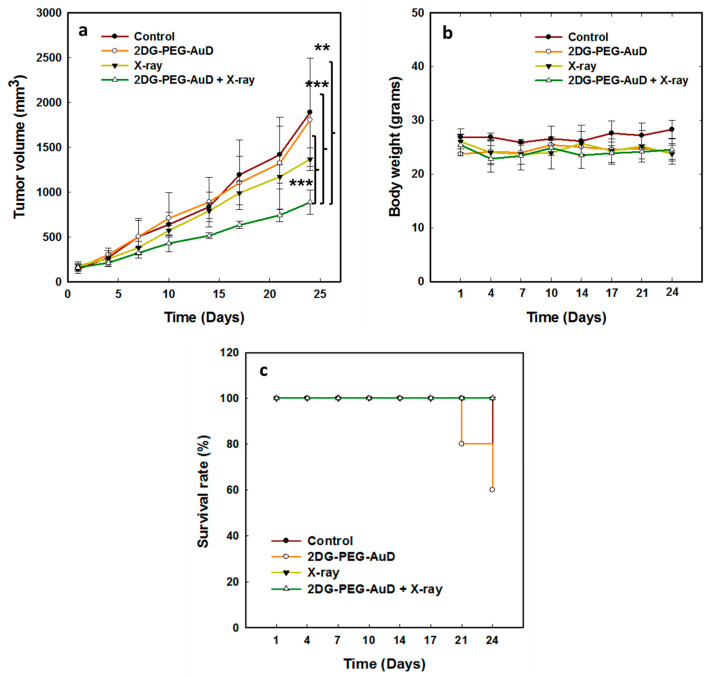
(**a**) In vivo therapeutic efficacy of 2DG-PEG-AuD combined with or without 8 Gy irradiation (four fractions of 2 Gy every day) in nude mice bearing LS174T tumors. Each value represents the mean ± SEM of tumor volume in mm^3^ relative to that measured at the beginning of treatment. (**b**) The body weight of mice undergoing various treatments. (**c**) Mouse survival following various treatments. Error bars represent standard deviation for five animals per group. Asterisks (**, ***) denote statistically significant differences (*p* < 0.01), and (*p* < 0.001) calculated by Student’s *t*-test.

We evaluated the in vivo biodistribution of 2DG-PEG-AuD in tumor-bearing mice and compared with PEG-AuD. The accumulation in tumor and vital organs was quantified by using an inductively coupled plasma mass spectrometer (ICPMS, Perkin Elmer, NexION 2000) 24 h after their i.v. administration. The accumulated Au was expressed in mg/g. The results showed that 2DG-PEG-AuD was mostly accumulated in mononuclear phagocytic organs, such as liver (17.01) and spleen (14.32), followed by the accumulation in the tumor (5.388). 2DG-PEG-AuD showed accumulation in other organs, such as kidney (2.491), heart (1.472), and lungs (6.376). Compared to tumor-targeted 2DG-PEG-AuD, PEG-AuD showed relatively less accumulation in the tumor (2.148) due to the lack of tumor targeting and exhibited major accumulation in liver (10.65) and spleen (13.84), followed by kidney (1.291), heart (1.208), and lungs (3.198). The in vitro results encouraged us to evaluate the in vivo radiosensitizing potential of 2DG-PEG-AuD in tumor-bearing mice models (Figure 11a). For this study, treatments were divided into four groups: control; 2DG-PEG-AuD without X-ray; X-ray only; and 2DG-PEG-AuD with X-ray. When tumor volume reached approximately 150 mm^3^, mice were subjected to various treatments and tumor volume was monitored for 24 d. The control and 2DG-PEG-AuD without X-ray irradiation group clearly showed no effect on tumor growth. However, an approximately 13- and 12-fold increase in tumor volume was observed for the control and 2DG-PEG-AuD group, respectively. The mice group treated with X-rays only exhibited tumor inhibition from day 7 and showed an approximately 1.3-fold tumor inhibition when compared to the control group on day 24. The 2DG-PEG-AuD + X-ray combination treatment group demonstrated superior tumor inhibition when compared to the rest of the treatment groups. Specifically, tumor inhibition was obvious from day 4, continued to inhibit throughout the entire study, and the tumor inhibition rate was found to be approximately 13.9-fold when compared to that of the control group. The enhanced therapeutic effect observed was due to the ability of 2DG-PEG-AuD to sensitize X-rays to destroy the cancer cells. In addition, the body weight of the mice undergoing various treatments did not change throughout the entire study period, which indicates that the treatment was safe, and no toxicity was induced (Figure 11b). In a period of 24 d after the treatment, all of the mice in the X-ray only and 2DG-PEG-AuD + X-ray groups survived. However, survival decreased to 80% at day 21, and further reduced to 60% at day 24 in the 2DG-PEG-AuD-treated group, because mice were removed from the treatment due to an increase in tumor volume over 2000 mm^3^ (Figure 11c). Similarly, survival decreased to 80% on day 24 in the X-ray treatment group for the same reason.

Theranostic nanomedicine has the immense potential to revolutionize cancer therapy. The targeted 2DG-PEG-AuD demonstrated excellent CT imaging contrast both in vitro and in vivo. The potential of PEG-AuD as a radiosensitizer was displayed through their ability to produce various ROS and damage to cellular components, such as DNA. Remarkably, the copious production of ROS by 2DG-PEG-AuD-mediated RT drove the cancer cell death pathway towards ferroptosis. Finally, in vivo studies demonstrated the theranostic capability of our synthesized 2DG-PEG-AuD which generated high-resolution and functional CT images of tumors and inhibited tumor growth.

## 4. Conclusions

In aggregate, we have successfully synthesized PEG-AuD and further modified with 2DG to impart tumor-targeting functions. Significant CT contrast enhancements were achieved using both in vitro and in vivo studies. Results from this work indicate that 2DG-PEG-AuD could greatly potentiate CT imaging capability in cancer diagnostics by providing high-resolution anatomical and functional images in a single CT scan. We also showed the ability of our targeted nanosytem to produce various biologically important ROS in the presence of X-rays. The surplus ROS generated damaged the DNA and instigated the ferroptosis pathway. In addition, our nanodots showed the ability to radiosensitize the tumor, and demonstrated therapeutic functions in the presence of X-rays. Therefore, 2DG-PEG-AuD developed in this study could be a potential candidate as an effective theranostic agent in clinical applications.

## Figures and Tables

**Table 1:** DLS and zeta potential of AuD, AuD-PEG, and 2DG-PEG-AuD.

Characterization	AuD	PEG-AuD	2DG-PEG-AuD
DLS	11 nm	14 nm	20 nm
Zeta potential	−31 mV	−24 mV	−11 mV

**Table 2:** Concentration of AuD and corresponding HU units from Figure 3.

Concentration of AuD (mg/mL)	Hounsfield Units (HU)
1	110
3	250
5	340
10	750
30	1850
60	3200
80	4700

## Data Availability

The data presented in this study are available on request from the corresponding author.
